# Keeping the rhythm: light/dark cycles during postharvest storage preserve the tissue integrity and nutritional content of leafy plants

**DOI:** 10.1186/s12870-015-0474-9

**Published:** 2015-03-27

**Authors:** John D Liu, Danielle Goodspeed, Zhengji Sheng, Baohua Li, Yiran Yang, Daniel J Kliebenstein, Janet Braam

**Affiliations:** Department of BioSciences, Rice University, Houston, TX 77005 USA; Department of Plant Sciences, University of California, Davis, CA 95616 USA; DynaMo Centre of Excellence, Department of Plant and Environmental Sciences, Faculty of Science, University of Copenhagen, Thorvaldsensvej 40, 1871 Frederiksberg C, Denmark; Current Address: Department of Obstetrics and Gynecology, Baylor College of Medicine, Houston, TX 77030 USA

**Keywords:** Biological clock, Chlorophyll, Circadian clock, Circadian rhythms, Vegetable and fruit preservation, Diurnal, Glucosinolates, Nutritional value, Vegetable and fruit shelf life

## Abstract

**Background:**

The modular body structure of plants enables detached plant organs, such as postharvest fruits and vegetables, to maintain active responsiveness to environmental stimuli, including daily cycles of light and darkness. Twenty-four hour light/darkness cycles entrain plant circadian clock rhythms, which provide advantage to plants. Here, we tested whether green leafy vegetables gain longevity advantage by being stored under light/dark cycles designed to maintain biological rhythms.

**Results:**

Light/dark cycles during postharvest storage improved several aspects of plant tissue performance comparable to that provided by refrigeration. Tissue integrity, green coloration, and chlorophyll content were generally enhanced by cycling of light and darkness compared to constant light or darkness during storage. In addition, the levels of the phytonutrient glucosinolates in kale and cabbage remained at higher levels over time when the leaf tissue was stored under light/dark cycles.

**Conclusions:**

Maintenance of the daily cycling of light and dark periods during postharvest storage may slow the decline of plant tissues, such as green leafy vegetables, improving not only appearance but also the health value of the crops through the maintenance of chlorophyll and phytochemical content after harvest.

**Electronic supplementary material:**

The online version of this article (doi:10.1186/s12870-015-0474-9) contains supplementary material, which is available to authorized users.

## Background

Approximately one-third of food produced globally is lost or wasted [1], yet fewer resources are devoted to postharvest research and development than to efforts for improving productivity [2]. The modular design of plants [3] allows plant tissues and organs to remain biologically active even after harvest [4,5]. Therefore, capitalizing on the ability of harvested vegetables and fruits to continue to sense and respond to diverse stimuli, similarly to intact plants, may be a powerful approach to promote postharvest quality.

Research demonstrating the biological advantage of a functional circadian clock in plants led us to investigate whether maintaining diurnal (24-hour light/dark) cycles may promote longevity and therefore reduced yield loss during postharvest storage of vegetables. The circadian clock enables plants to anticipate and prepare for the daily environmental changes that occur as a consequence of the rotation of the earth. Coordination of plant circadian rhythms with the external environment provides growth and reproductive advantages to plants [6], as well as enhanced resistance to insects [7] and pathogens [8,9]. The circadian clock also regulates aspects of plant biology that may have human health impact, such as levels of carbohydrates [10], ascorbic acid [11], chlorophyll [12], and glucosinolates [5] in edible plant species.

Plants exhibit exquisite sensitivity to light stimuli, and isolated plant leaves maintain responsiveness to light after harvest and can continue light-dependent biological processes, such as photosynthesis [13]. Additionally, the clocks of postharvest fruit and vegetable tissues can be entrained with 12-hour light/12-hour darkness cycles producing rhythmic behaviors not observed in tissues stored in constant light or constant dark [5]. A few studies have examined the effects of light on performance and longevity during postharvest storage [14]. For example, light exposure delays broccoli senescence and yellowing [13,15] but accelerates browning in cauliflower, a close relative of broccoli [16,17]. Other studies report that light exposure to broccoli during postharvest storage either provides no additional benefits [17] or decreases performance [18]. Postharvest light exposure improves chlorophyll content in cabbage [19], but leads to increased browning of romaine lettuce leaves [20]. Although exposure of spinach to light during postharvest storage can improve nutritional value [21,22], light can also accelerate spinach water loss, leading to wilting [22]. Together, these findings are inconclusive as to whether light exposure during postharvest storage can be generally beneficial, and the variation of the results may be attributable to differences in the plant species examined and the specific conditions used during postharvest storage, such as lighting intensities, temperature, humidity or packaging. Alternatively, light may be advantageous but only if present in its natural context with 24-hour periodicity because of such timing on circadian clock function.

This study aimed to examine whether mimicking aspects of the natural environment predicted to maintain circadian biological rhythms during postharvest storage of green leafy vegetables improves performance and longevity compared to postharvest storage under constant light or constant darkness. We focused this work on several popular and nutritionally valuable species, including kale (*Brassica oleracea* cv. acephala group) and cabbage (*Brassica oleracea*), members of the Brassicaceae family with worldwide production of approximately 70 million tons [23]. In addition, we analyzed green leaf lettuce (*Lactuca sativa*) and spinach (*Spinacia oleracea*), which have worldwide production of approximately 25 and 22 million tons, respectively [23]. Here, we report on the promotion of postharvest longevity, including tissue integrity and nutritional value, of green leafy vegetables by provision of 24-hour light/dark cycles during storage compared to storage under constant light or constant darkness.

## Methods

### Plant materials and storage conditions

Kale (*Brassica oleracea* cv. acephala group), cabbage (*Brassica oleracea*), green leaf lettuce (*Lactuca sativa*) and spinach (*Spinacia oleracea*) were purchased from a local organic grocer. Leaf tissue was cut into 2 cm disks and placed on 0.5% agar as described previously [5]. Light-treated leaf disks were stored under 120 ± 10 μE · m^−2^ · s^−1^ (E = Einstein, defined as one mole of photons) at 22°C for either cycles of 12 hours of light followed by 12 hours of darkness or constant 24-hour light. Leaf disks stored in constant darkness were either maintained at 22°C or refrigerated at 4°C. Samples for analysis were collected 6 hours after initiation of the light period for samples stored under 12-hour light/12-hour dark cycles or at comparable times for samples stored in constant light, in constant darkness, or under refrigeration to avoid time-of-day dependent differences in measured values.

### Chlorophyll measurements

Frozen leaf disks were ground using a mortar and pestle, and approximately 50 mg of the resulting powder was mixed with 0.5 mL of 80% (v/v) acetone and incubated overnight at 4°C. Samples were centrifuged at 14,000 x g for 10 minutes at 4°C. The absorbance of the supernatant was measured spectrophotometrically at 645 and 663 nm using a Tecan Infinite M200 PRO (Tecan, Morrisville, NC). Chlorophyll concentrations relative to fresh weight were determined using the formula: total chlorophyll (μg/g fresh weight) = (17.76A_645_ + 7.34A_663_)/g plant tissue, as described [24]. Chlorophyll concentrations per gram dry weight were determined after plant materials were freeze dried at −80°C at 0.04 mBar overnight using a FreeZone 4.5 liter benchtop freeze dry system (Labconco, Kansas City, M.O.).

### Electrolyte leakage measurements

For electrolyte leakage measurements, 2 cm leaf disks were placed into 50 ml of deionized room temperature water. After 30 minutes, electrical conductivity was measured using a Horiba B-173 Twin Cond Conductivity Meter (Horiba Instruments Inc., Kyoto, Japan). To avoid complications associated with electrolyte leakage due to initially cutting out the leaf disks, the first electrolyte leakage measurements were delayed as specified in the text.

### Glucosinolate measurements

Tissue frozen with liquid nitrogen was ground using a mortar and pestle and then submerged in 90% methanol and extracted. Glucosinolates were identified and quantified in comparison to reference standards using HPLC-DAD, as previously described [25]. Total glucosinolate levels were determined by totaling the concentrations of individually quantified glucosinolate species (Additional file 1: Figure S1 and S2) as described previously [5].

### Statistical analyses

Data were subjected to analysis of variance (ANOVA) with Bonferroni Post Hoc analysis to examine differences between leaf tissue stored under light/dark cycles to those stored under alternative condition such as constant light, constant dark or refrigerated in constant dark at each time point using SPSS Statistics software. Comparisons of means between the beginning and end of the experiment were analyzed by Student’s *t*-test using SPSS Statistics software.

## Results

### Kale, cabbage, lettuce and spinach leaf tissues show improved appearance when stored under 12-hour light/12-hour dark cycles

**Figure 1:**
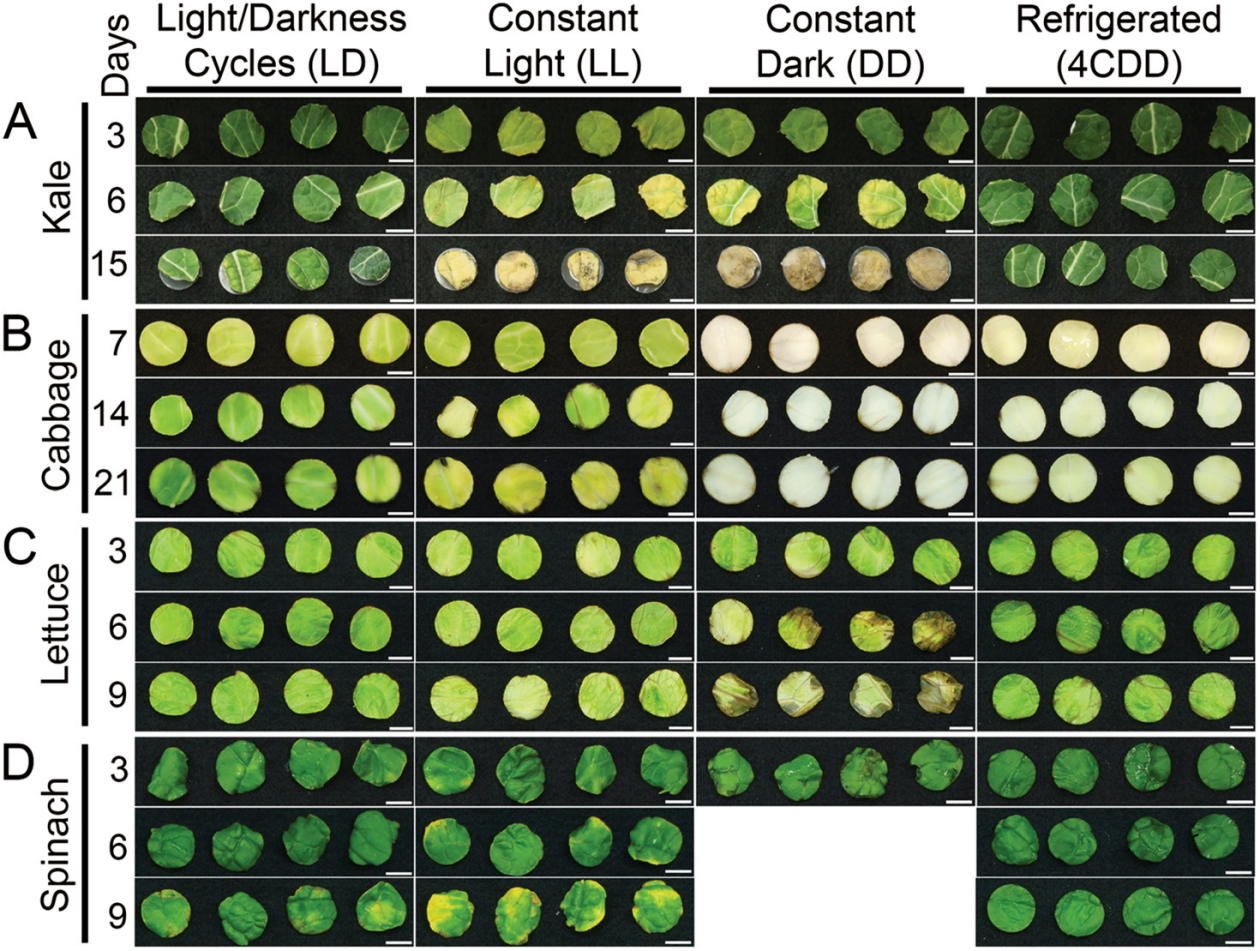
**Leaf disk appearance depends upon light exposure during post harvest storage.** Representative photographic images of kale **(A)**, cabbage **(B)**, lettuce **(C)**, and spinach **(D)** leaf disks after varying number of days (number of days indicated at left) of storage at 22°C under 12-hours light/12-hours darkness (Light/Dark Cycles, LD), constant light (LL), or constant dark (DD), or at 4°C under constant dark (Refrigerated, 4CDD). Spinach disks disintegrated after 3 days when stored under constant darkness and therefore images were not available. (Scale bars, 1 cm).

Fruits and vegetables after harvest can respond to repeated cycles of 12-hour light/12-hour dark, resulting in circadian clock function and rhythmic behaviors [5]. Because a functional plant circadian clock is physiologically advantageous [7,26,27] we sought to address whether postharvest storage under conditions that simulate day/night cycles, thereby potentially maintaining biological rhythms, would affect postharvest longevity. We chose to address this question using green leafy vegetables, including commonly consumed kale (*Brassica oleracea* cv. acephala group), cabbage (*Brassica oleracea*), green leaf lettuce (*Lactuca sativa*) and spinach (*Spinacia oleracea*), because we anticipated that the leaf organ would likely maintain light sensitivity and responsiveness even after harvest. To begin to determine whether daily light/dark cycles during postharvest storage affects leaf longevity, we compared the overall appearance of leaf disks that were stored at 22°C under cycles of 12-hour light/12-hour darkness (LD) versus leaf disks stored under constant light (LL) or constant darkness (DD) for various lengths of time (Figure 1).

Under cycles of 12-hour light/12-hour darkness, kale leaf disks were dark green after 3 days of storage (Figure 1A; LD). After 6 days and 15 days of storage, the kale disks showed lighter green coloration than the kale disks stored for 3 days (Figure 1A; LD). However, the kale leaf disks stored under constant light were lighter green than the kale disks stored under light/dark cycles and showed some brown or yellow discoloration after 3 and 6 days (Figure 1A; LL). By 15 days, the kale leaf disks stored under constant light lost nearly all green coloration and showed light and dark shades of browning with shape changes resulting from leaf folding and shrinkage (Figure 1A; LL). The kale leaf disks stored under constant darkness resembled those stored under constant light, except that the 3-day kale samples were darker green than the 3-day constant light-stored kale leaf disks (Figure 1A; DD), suggesting that the constant light may have constituted a greater stress on the kale leaves than constant darkness. These results indicate that postharvest storage with daily cycling of light and darkness improved the appearance of the kale leaf tissue compared to storage under either constant light or constant darkness. However, the preservation benefit obtained from postharvest storage under light/dark cycles at 22°C appeared to be less than that provided by refrigeration; kale leaf disks stored at 4°C with constant darkness, were comparable in their dark green coloration whether stored for 3, 6 or 15 days (Figure 1A; 4CDD).

Cabbage leaf disks stored under cycles of 12-hour light/12-hour darkness showed brown spots along the disk edges that increased in intensity over the storage period of 7, 14, and 21 days (Figure 1B; LD). However, although the 7-day cabbage leaf samples were light green in coloration, the 14- and 21-day cabbage leaf disks stored under light/dark cycles had darker green coloration (Figure 1B; LD), suggesting increased photosynthetic activity over storage time. In contrast, although the cabbage leaf disks stored under constant light were also light green after 7 days of storage, the 14- and 21-day cabbage leaf disks were more yellow and included more brown discolorations (Figure 1B; LL). Remarkably, the absence of light exposure during post-harvest storage had a dramatic effect on the cabbage leaf disk coloration. Cabbage leaf disks stored under constant darkness at either 22°C or 4°C were pale tan or yellow after 3 days of storage (Figure 1B; DD and 4CDD). The constant darkness-exposed cabbage leaf disks stored at 22°C appeared nearly white in color by 14 and 21 days; those at 4°C had a yellowish appearance after 2 or 3 weeks of storage (Figure 1B; DD and 4CDD).

Lettuce and spinach leaf disks tissue were nearly uniformly green, with little difference in color intensity between 3 and 6 days of storage under cycles of 12-hour light/12-hour darkness (Figure 1C,D; LD). By 9 days of storage under light/dark cycles, however, both lettuce and spinach leaf disks looked slightly less green, and most of the spinach leaf disks had distinct patches of yellow (Figure 1C,D; LD). In contrast, the loss of green coloration and increased yellowing over time was much more apparent in the lettuce and spinach leaf disks stored under constant light; the lettuce leaf disks were pale green by 9 days (Figure 1C; LL), and all the spinach disks had large yellow patches (Figure 1D; LL). Lettuce and spinach leaf disks stored under constant darkness displayed small brown patches by 3 days (Figure 1C,D; DD). After 6 and 9 days of storage under constant darkness, the lettuce disks had large wet patches of darkened tissue (Figure 1C; DD). However, the spinach leaf disks stored under constant darkness at 22°C for 6 days completely disintegrated and therefore could not be moved for photographic imaging. Lettuce and spinach leaf disks stored in constant darkness at 4°C largely maintained dark green coloration at 6 days and were lighter green at 9 days, similar to that of the disks stored under light/dark cycles at 22°C (Figure 1C,D; 4CDD). However, after 6 days of storage at 4°C, the lettuce leaf disks also displayed browning around the vascular tissues (Figure 1C; 4CDD).

Overall, the image analysis shown in Figure 1 suggests that postharvest storage under cycles of 12-hour light and 12-hour darkness may enable kale, cabbage, lettuce, and spinach leaf tissues to maintain physiological functioning for longer durations after harvest. The reduction in green color and appearance of brown discoloration suggests that postharvest storage in constant light or constant darkness may accelerate loss of tissue viability.

### Chlorophyll content retention is higher in leaf tissue stored under cycles of 12-hour light/12-hour darkness

**Figure 2:**
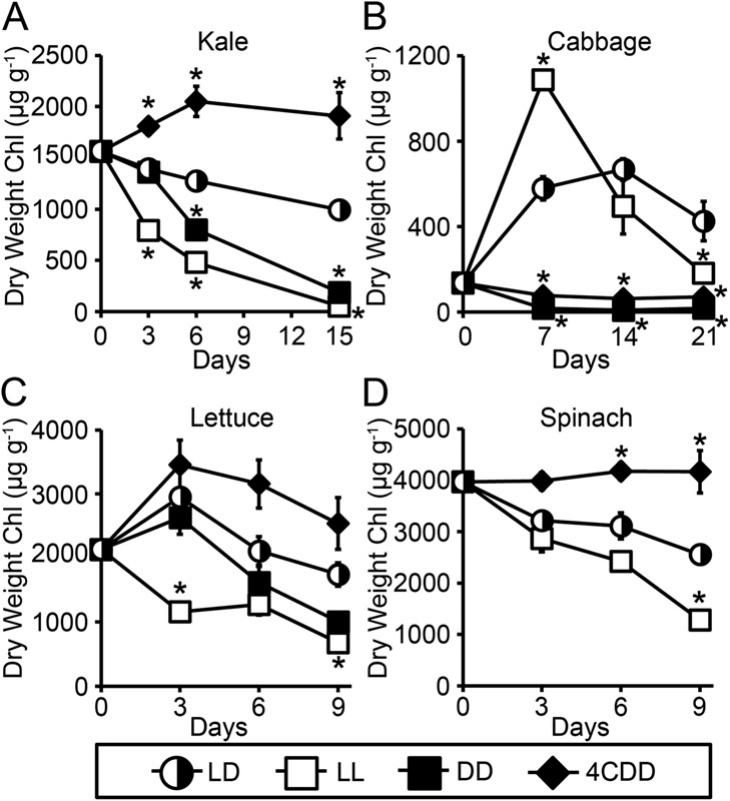
**Chlorophyll content retention was higher in kale, cabbage, lettuce and spinach leaves stored in light/dark cycles compared to constant light or constant darkness at 22°C.** Chlorophyll content relative to dry weight was quantified from leaf tissue disks of kale **(A)**, cabbage **(B)**, lettuce **(C)** and spinach **(D)** stored under cycles of 12-hours light/12-hours darkness (LD, half-filled circles), constant light (LL, open squares), or constant darkness (DD, filled squares) at 22°C, or under constant darkness at 4°C (4CDD, filled diamonds). Mean ± SE; n = 4. Asterisks indicate significant differences (p < 0.05. ANOVA Test with Bonferroni Post Hoc analysis) between data derived from leaf disks stored under light/dark cycles (22°C) and that derived from leaf disks stored under other conditions at each time point. Statistical analyses of differences over time for each plant type are shown in Additional file 1: Figure S4.

To further characterize kale, cabbage, lettuce and spinach leaf health and viability during postharvest storage, we quantified chlorophyll content in leaf samples after storage under cycles of 12-hour light/12-hour darkness to leaf tissues stored under constant light or constant darkness. Three sets of comparative data are shown (Figure 2, Additional file 1: Figure S3 and Figure S4). Because our primary focus was to determine whether light/dark cycles were advantageous relative to constant light or constant dark storage conditions, we first conducted two-way comparative statistical analyses between data derived from the samples stored under light/dark cycles relative to comparable samples stored under the alternative condition (that is, constant light, constant dark or refrigerated/dark). Figure 2 presents statistical analysis of storage-dependent differences in chlorophyll levels relative to dry weight at each time point. Additional file 1: Figure S3 shows similar analyses but of storage-dependent differences in chlorophyll levels relative to fresh weight. Finally, to evaluate whether there were significant changes in chlorophyll levels of each plant type over time, statistical analyses of differences in chlorophyll content at the beginning and end of the experiments for kale, cabbage, lettuce, and spinach are shown in Additional file 1: Figure S4.

Consistent with the loss of green coloration in the representative leaf disk samples shown in Figure 1A, postharvest storage of kale leaf disks in either constant light or constant dark led to significantly greater losses in kale chlorophyll content within 3 or 6 days of postharvest storage compared to storage under 12-hour light/12-hour dark cycles (Figure 2A; Additional file 1: Figure S3A). Kale leaf disks stored for 15 days under constant light lost 97% and 93% of their original chlorophyll content relative to dry and fresh weight, respectively (Additional file 1: Figure S4A, E); kale leaf disks stored under constant darkness lost 88% and 89% of their chlorophyll content relative to total dry and fresh weight, respectively (Additional file 1: Figure S4A, E). In contrast, 15 days of storage under cycles of 12-hour light/12-hour darkness led to loss of only 36% and 9% of the kale leaf disk chlorophyll relative to dry and fresh weight, respectively (Additional file 1: Figure S4A, E). Kale leaf disks stored at 4°C under constant darkness, however, performed statistically better than those stored under cycles of light/dark (Figure 2A; Additional file 1: Figure S3A), with no significant decreases in chlorophyll content relative to total dry or fresh weight over the full 15 days of the experiment (Additional file 1: Figure S4A, E).

Postharvest storage of cabbage leaf disks under light/dark cycles resulted in significantly higher chlorophyll levels than storage under constant dark either at 22°C or 4°C at all time points examined (Figure 2B; Additional file 1: Figure S3B). Indeed, cabbage leaf disks began with only modest chlorophyll levels (Figure 2B; Additional file 1: Figure S3B). However, when the cabbage leaf disks were stored under either constant light or light/dark cycles, chlorophyll content increased over time (Figure 2B, Additional file 1: Figure S3B) with significantly higher levels remaining even after three weeks of storage (Additional file 1: Figure S4B, F). Light-induced synthesis is likely responsible for the elevated chlorophyll levels (Figure 2B; Additional file 1: Figure S3B, S4B, F) and the enhanced green coloration observed in the cabbage leaf images (Figure 1B) of the samples stored under light/dark cycles or constant light but absent in the cabbage leaf disks stored under constant darkness either at 22°C or 4°C (Figure 1B, 2B; Additional file 1: Figure S3B). Storage under light/dark cycles was also more successful than constant light exposure in maintaining higher chlorophyll levels after long-term storage of 3 weeks (Figure 2B; Additional file 1: Figure S3B). Over time, storage under constant light may be counterproductive; whereas cabbage leaf disks stored for 7 days under constant light had significantly higher chlorophyll content than leaf disks stored under light/dark cycles (Figure 2B; Additional file 1: Figure S3B), by three weeks of storage, the leaf disks stored under light/dark cycles retained at least 2-fold more chlorophyll than samples stored under constant light (Figure 2B; Additional file 1: Figure S3B). These results indicate that light during postharvest storage can have a profound effect on chlorophyll levels in cabbage, consistent with previous reports [19], and that diurnal cycling of light and darkness prolongs this benefit during longer term storage.

Storage under cycles of 12-hour light/12-hour darkness also promoted chlorophyll retention (relative to both dry and fresh weight) in lettuce leaf disks, comparable to that of lettuce leaf disks stored under refrigeration; chlorophyll levels were statistically indistinguishable between lettuce leaf disks stored under light/dark cycles versus those refrigerated under constant darkness conditions after 3, 6 or 9 days of storage (Figure 2C; Additional file 1: Figure S3C). Postharvest storage of lettuce leaf disks either at 22°C under light/dark cycles or under refrigeration resulted in no significant change in chlorophyll content over the course of the 9-day experiment, whereas the lettuce leaf disks stored under either constant light or constant darkness, lost more than 50% of their starting chlorophyll content (Additional file 1: Figure S4C, G).

Chlorophyll content of spinach leaf disks was not significantly affected by treatment conditions for the first 3 days of postharvest storage (Figure 2D; Additional file 1: Figure S3D, S4D, H). However, the spinach leaf disks stored at 22°C in constant darkness disintegrated by 6 days and were therefore unable to be further analyzed. Spinach leaf disks stored under light/dark cycles had similar chlorophyll content to those stored under constant light with relatively stable chlorophyll retention until day 9 when chlorophyll levels in both samples decreased significantly from initial levels (Figure 2D; Additional file 1: Figure S3D, S4D, H). In contrast, refrigeration led to stable chlorophyll levels in the spinach leaf disks over the course of the experiment (Additional file 1: Figure S4D, H).

These results indicate that chlorophyll content of postharvest green leafy vegetables varies depending upon the storage conditions and suggests that storage under 12-hour cycles of light and darkness, known to maintain the plant circadian clock [5], can improve kale, cabbage and lettuce chlorophyll content maintenance relative to storage in constant light or constant dark. Perhaps surprisingly light/dark cycles during postharvest storage may be at least as beneficial as refrigeration with respect to chlorophyll content for cabbage and lettuce.

### Tissue integrity improved by postharvest storage under light/dark cycles

**Figure 3:**
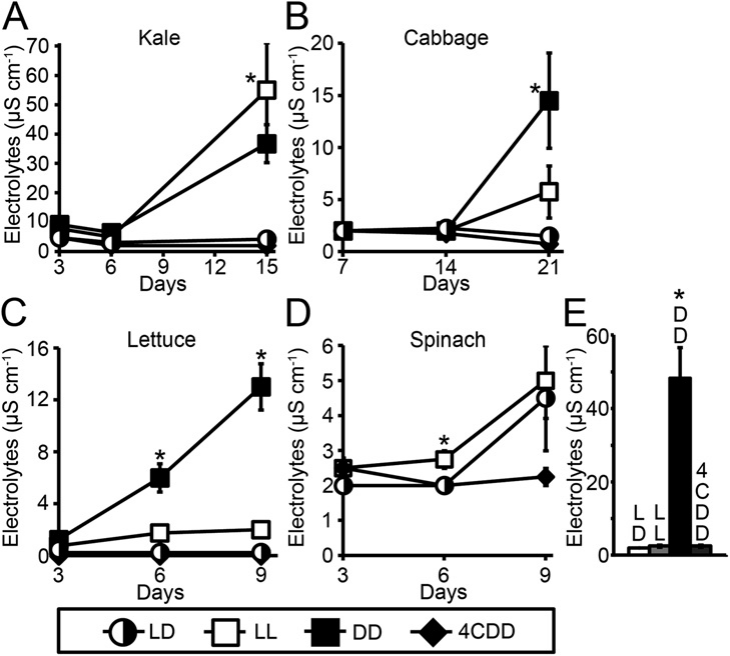
**Electrolyte leakage from kale, cabbage, lettuce, and spinach leaf disks was affected by light exposure during storage.** Electrolytes released from leaves of kale **(A)**, cabbage **(B)**, lettuce **(C)** or spinach **(D & E)** were measured after storage under cycles of 12-hour light/12-hour darkness (LD, half-filled circles), constant light (LL, open squares), or constant darkness (DD, filled squares) at 22°C or under constant darkness at 4°C (4CDD, filled diamonds). Mean ± SE; n = 4. Asterisks indicate significant differences (p < 0.05, ANOVA Test with Bonferroni Post Hoc analysis) between data derived from leaf disks stored under light/dark cycles (22°C) and that derived from leaf disks stored under other conditions at each time point. Statistical analyses of differences over time for each plant type are shown in Additional file 1: Figure S5.

Over time during postharvest storage, plant tissues typically show visible signs of tissue disintegration (e.g., Figure 1). To determine if maintaining light/dark cycles during storage of post harvest leafy vegetables could prolong tissue integrity, we compared electrolyte leakage from kale, cabbage, lettuce, and spinach leaf disks stored over time under cycles of 12-hour light/12-hour darkness to leaf disks stored under constant light or constant darkness at 22°C or constant darkness at 4°C. Figure 3 shows that postharvest storage under light/dark cycles and refrigeration were comparable, with respect to leaf tissue integrity maintenance of kale, cabbage and lettuce, as measured by electrolyte leakage, (Figure 3A-C; LD and 4CDD). When directly comparing light/dark storage to other conditions, a statistically significant benefit to diurnal stimuli during storage was apparent relative to constant light for kale (Figure 3A; LL), constant darkness for cabbage and lettuce (Figure 3B,C; DD), and constant darkness (at 3 days) and constant light (at 6 days) for spinach (Figure 3D; LL, and Figure 3E; DD).

Postharvest storage under constant dark was detrimental to kale, cabbage, and lettuce tissue integrity, with at least 4-fold increases in electrolyte leakage, whereas storage under light/dark cycles at 22°C or refrigeration resulted in no significant increase in electrolyte leakage over the course of the experiment (Additional file 1: Figure S5). Constant light treatment also led to significant increases in electrolyte leakage from kale and lettuce leaf disks, but not cabbage leaf disks, over the storage periods examined (Additional file 1: Figure S5).

Overall the results shown in Figure 3 and Additional file 1: Figure S5 provide evidence that daily cycles of light and darkness during postharvest storage resulted in superior leaf tissue integrity maintenance largely comparable to refrigeration, whereas either constant light or constant dark storage conditions were detrimental.

### Total glucosinolate levels are maintained when kale and cabbage are stored in light/dark cycles

Our results indicate that storage of kale, cabbage, lettuce, and spinach in light/dark cycles can improve the postharvest longevity of chlorophyll levels and tissue integrity. Next we were interested in determining whether plant maintenance under daily cycles of light and darkness affects human-health relevant metabolite content. In particular, we sought to examine whether kale and cabbage stored under light/dark cycles maintain their glucosinolate content longer than when stored under constant light, constant darkness, or refrigeration.

**Figure 4:**
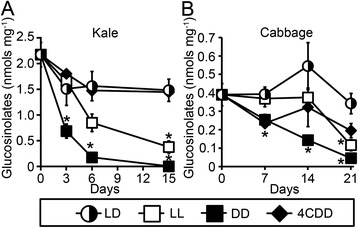
**Maintenance of total glucosinolate levels in kale and cabbage leaves when stored under light/dark cycles.** Glucosinolate species was quantified from leaf disks of kale **(A)** and cabbage **(B)** stored under cycles of 12-hour light/12-hour darkness (LD, half-filled circles), constant light (LL, open squares), or constant darkness (DD, filled squares) at 22°C or under constant darkness at 4°C (4CDD, filled diamonds). Mean ± SE; n = 4. Asterisks indicate significant differences (p < 0.05. ANOVA Test with Bonferroni Post Hoc analysis) between samples kept in light/darkness cycles (22°C) and those stored in constant light (22°C), constant dark (22°C) and under constant darkness (4°C) for a specified time point. Statistical analyses of differences over time for each plant type are shown in Additional file 1: Figure S6.

Figure 4A shows total glucosinolate levels in kale leaf disks after 0, 3, 6 and 15 days of postharvest storage under different conditions. Individual glucosinolate levels are shown in Additional file 1: Figure S1. Total glucosinolate levels were comparable between kale leaf disks stored at 22°C under light/dark cycles and leaf disks stored at 4°C in the dark (Figure 4A); after 15 days of postharvest storage under these conditions, total glucosinolate levels decreased by less than 35% (Additional file 1: Figure S6A). In comparison to light/dark storage conditions, constant light or constant darkness exposure during storage resulted in significantly reduced glucosinolate content in the kale leaf disks (Figure 4A). By 15 days of postharvest storage under constant light or constant darkness at 22°C, the kale leaf disks lost over 80% and 99% of initial levels, respectively.

Daily cycles of light and darkness also promoted maintenance of glucosinolate content during postharvest storage of cabbage (Figure 4B; Additional file 1: Figure S2). Total glucosinolate content in the cabbage leaf disks stored under light/dark cycles remained stable with no significant fluctuation in levels over the 21 days of analysis (Figure 4B; Additional file 1: Figure S6B). In comparison to total glucosinolate levels in light/dark-stored cabbage, the glucosinolate levels were significantly lower by 7 days when cabbage leaf disks were stored under constant darkness (Figure 4B; DD) and by 21 days when stored under constant light (Figure 4B; LL). Total glucosinolate levels declined by 70% and 88%, respectively, in cabbage disks stored at 22°C under constant light or constant darkness (Additional file 1: Figure S6B). Remarkably, glucosinolate levels of the cabbage leaf disks stored at 4°C also showed a significant decrease by 21 days, with a loss of 50% of the initial glucosinolate levels (Additional file 1: Figure S6B), indicating that storage under cycle of light/darkness led to enhanced retention of this valuable phytochemical even relative to refrigeration.

## Discussion

In this work, we examined whether kale, cabbage, lettuce and spinach leaf tissue maintain the ability to respond to light/dark cycles during postharvest storage and whether under these conditions that better mimic the natural light cycles of the environment tissue deterioration would be reduced. Our goal was to expose plant tissues to diurnal conditions known to maintain the functioning of the circadian clock [5] and thereby capitalize on physiological enhancements conferred by robust circadian rhythms. Plants grown under light/dark cycles that match the endogenous cycling of their internal circadian clock have a growth and reproductive advantage over plants exposed to light/dark cycles that do not match their internal oscillator [26,28]. Furthermore, phasing of circadian rhythms so as to be synchronized with the external environment promotes biotic stress resistance [5,7,27].

We found that storing green leafy vegetables in cycles of 12 hours of light followed by 12 hours of darkness improved several postharvest performance markers compared to postharvest storage of the leaf tissues under constant light or constant darkness. Similarly, a modest reduction in senescence was noted for post-harvest broccoli stored under natural light/dark cycles [15]. Perhaps surprisingly, we found that storage in light/dark cycles resulted in several aspects of postharvest performance being comparable to storage under refrigeration, a commonly practiced method of postharvest storage thought to slow down cellular breakdown [2]. The longevity of kale and lettuce leaf color, chlorophyll levels, and tissue integrity, which are important contributors to the appeal of green leafy vegetables to consumers [29], were largely indistinguishable whether the kale and lettuce leaf samples were stored at 22°C under light/dark cycles or were stored under refrigeration in constant darkness (Figures 1 and 3). Spinach leaf samples also maintained green coloration and chlorophyll levels under light/dark cycles at 22°C as well as when refrigerated, but refrigeration was more successful at preventing spinach leaf tissue breakdown. Significant improvement of green coloration and chlorophyll content was seen when cabbage leaves were stored under light/dark cycles at 22°C compared to refrigeration, demonstrating that light may not only be important for clock entrainment but also can provide the additional benefit of promoting continued photosynthesis during postharvest storage. Promotion of photosynthesis and/or chlorophyll levels was previously observed in post-harvest crops stored under light [15,17,30]. However, constant light during post-harvest storage can also cause detrimental physiological activity, such as respiration leading to browning [30] and transpiration contributing to weight loss [16,18,31]. Therefore, cycling of light treatment with darkness periods may not only maintain clock function but may also avoid physiological damage that may occur in plant tissues under too much light.

In addition to improvement of green leafy vegetable appearance by postharvest storage under light/dark cycles, we found that this postharvest storage treatment of plant crops may improve human health benefits through maintenance of phytochemical content (Figures 2 and 4). Chlorophyll, responsible for the visual appeal of green leafy vegetables [29], also has beneficial impact upon human health upon ingestion. Chlorophyll can limit efficacy of carcinogens, such aflatoxin B_1_ [32-36] and can activate Phase II detoxifying enzymes [37]. Additional anticancer benefit may derive from glucosinolates in kale and cabbage. Glucosinolates, sulfur-containing compounds that play a major role in *Brassicaceae* plant herbivore defense [38], also underlie the human health benefits attributed to *Brassicaceae* (cruciferous) vegetable consumption [39,40]. For example, the glucosinolate glucoraphanin (4-methylsulfinylbutyl) has potent anticancer activity [37,41]. Previous studies have shown that glucosinolate levels can be maintained by refrigeration [42] or exposure to radiation [43]; here we find that post-harvest storage under light/dark cycles can also lead to sustained glucosinolate levels (Figure 4).

Light/dark cycles also maintain the circadian clock function of other edible crops after harvest, including zucchini, carrots, sweet potatoes, and blueberries [5]. These fruits and vegetables displayed time-dependent differences in insect resistance strongly suggesting temporal fluctuations in diverse metabolites, some of which may have important human health impact. Whether continued promotion of circadian periodicity postharvest can also improve longevity of tissue integrity and phytochemical content in diverse vegetables and fruits, as we have shown with kale, cabbage, lettuce, and spinach, remains to be investigated.

## Conclusions

Here we show that detached kale, cabbage, lettuce and spinach leaves show enhanced tissue longevity through continued exposure to diurnal light/dark cycles during storage. In addition, human-health relevant metabolites, such as glucosinolates and chlorophyll, are also retained at higher levels under diurnal storage conditions, suggesting that postharvest vegetables that retain natural rhythms during storage may be of greater nutritional value. These results provide additional evidence that, even postharvest, plant tissues retain the ability to sense external stimuli and respond in ways that affect tissue integrity and cellular metabolite levels. Translation of our understanding of plant physiology to postharvest crops, including the profound effects of the circadian clock on plant performance, may help to improve postharvest crop performance and reduce loss.

## Additional file

Additional file 1: Figure S1. Accumulation of individual glucosinolate species in kale disks. **Figure S2.** Accumulation of individual glucosinolate species in cabbage disks. **Figure S3.** Chlorophyll content in fresh weight leaf tissue was maintained at higher levels in light/darkness stored vegetables. **Figure S4.** Chlorophyll content in fresh and dry weight leaf tissue was maintained at higher levels over time when stored in light/darkness cycles or under refrigeration. **Figure S5.** Electrolyte leakage from kale, cabbage, lettuce, and spinach leaf disks is increased when stored under constant light or constant dark in kale, cabbage and spinach. **Figure S6.** Maintenance of total glucosinolate levels in kale and cabbage leaves when stored under light/dark cycles.

## Abbreviations

LD: 12-hour light/12-hour darkness cycles at 22°C
LL: 24-hour constant light at 22°C
DD: 24-hour constant dark at 22°C
4CDD: 24-hour constant dark under refrigeration at 4°C
