# Supplementary material for: Keeping the rhythm: light/dark cycles during postharvest storage preserve the tissue integrity and nutritional content of leafy plants
Source: BMC Plant Biol. 2015 Mar 27;15:92. doi: 10.1186/s12870-015-0474-9 (PMC4396971; doi:10.1186/s12870-015-0474-9)
Supplement: Additional file 1: Figure S1. — Accumulation of individual glucosinolate species in kale disks. Figure S2. Accumulation of individual glucosinolate species in cabbage disks. Figure S3. Chlorophyll content in fresh weight leaf tissue was maintained at higher levels in light/darkness stored vegetables. Figure S4. Chlorophyll content in fresh and dry weight leaf tissue was maintained at higher levels over time when stored in light/darkness cycles or under refrigeration. Figure S5. Electrolyte leakage from kale, cabbage, lettuce, and spinach leaf disks is increased when stored under constant light or constant dark in kale, cabbage and spinach. Figure S6. Maintenance of total glucosinolate levels in kale and cabbage leaves when stored under light/dark cycles. [file 12870_2015_474_MOESM1_ESM.pdf]

Keep the Clock Running: Light/Dark Cycles During Postharvest Storage Preserve the Tissue Integrity and Nutritional Content of Leafy Plants. John D Liu, Danielle Goodspeed, Zhengji Sheng, Baohua Li, Yiran Yang, Daniel J. Kliebenstein, and Janet Braam.

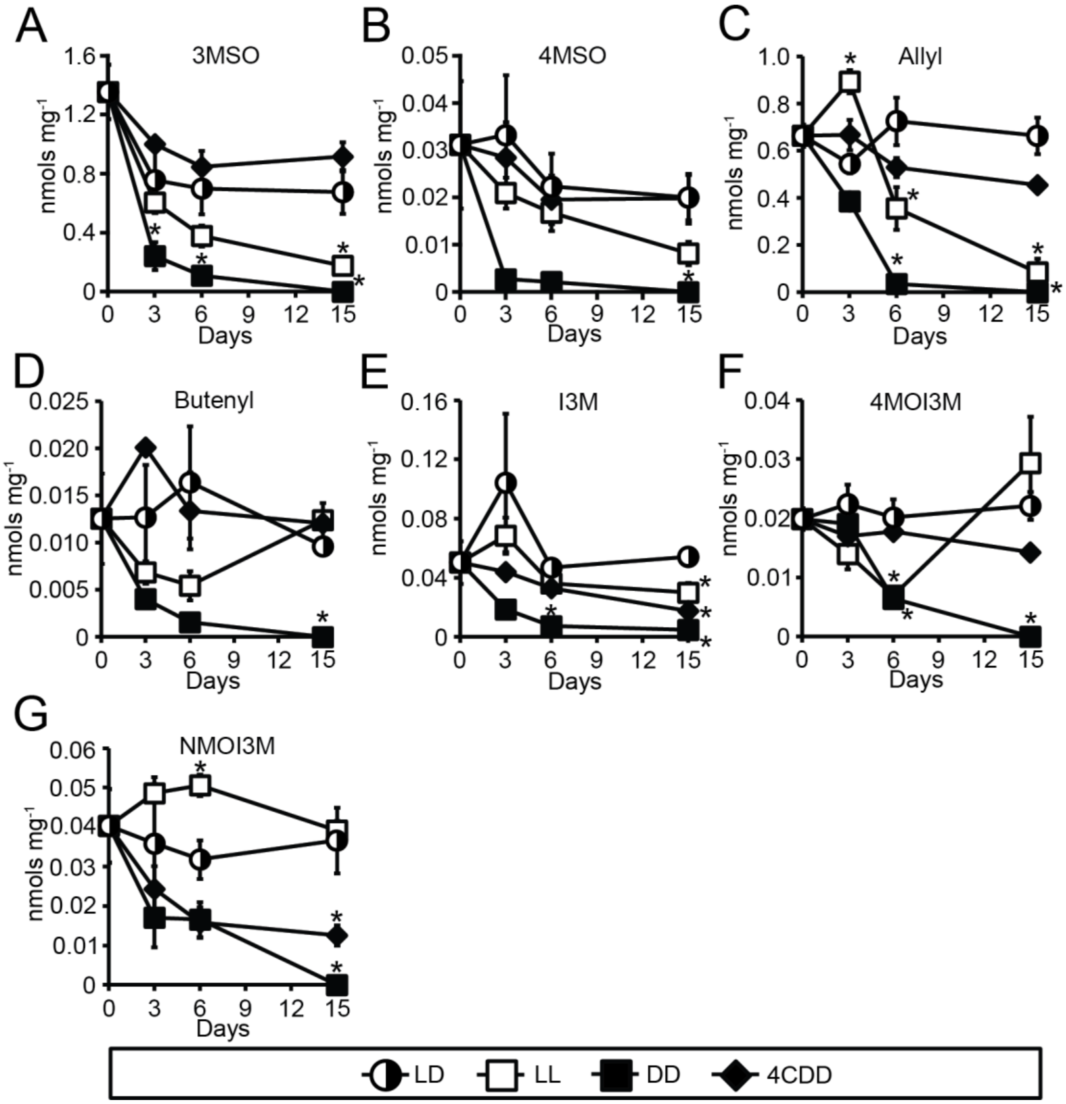

Figure S1. Accumulation of individual glucosinolate species in kale disks stored in 12 hour light/12 hour dark (22°C) (LD, half-filled circles), constant light (22°C) (LL, open squares); constant dark (22°C) (DD, filled squares), or refrigerated at 4°C in the dark (4CDD, filled diamonds). 3MSO, 3-methylsulfinylpropyl glucosinolate; 4MSO, 4-methylsulfinylbutyl glucosinolate; I3M, indoyl-3-methyl glucosinolate; 4MOI3M, 4-methoxy-indoyl-3-methyl glucosinolate; NMOI3M, 1-methoxy-indolyl-3-methyl glucosinolate. Mean ± SE; n = 4. Asterisks represent significant differences ($p<0.05$. ANOVA Test with Bonferroni Post Hoc analysis) between data derived from leaf disks stored under light/dark cycles (22°C) and that derived from leaf disks stored under other conditions at each time point.

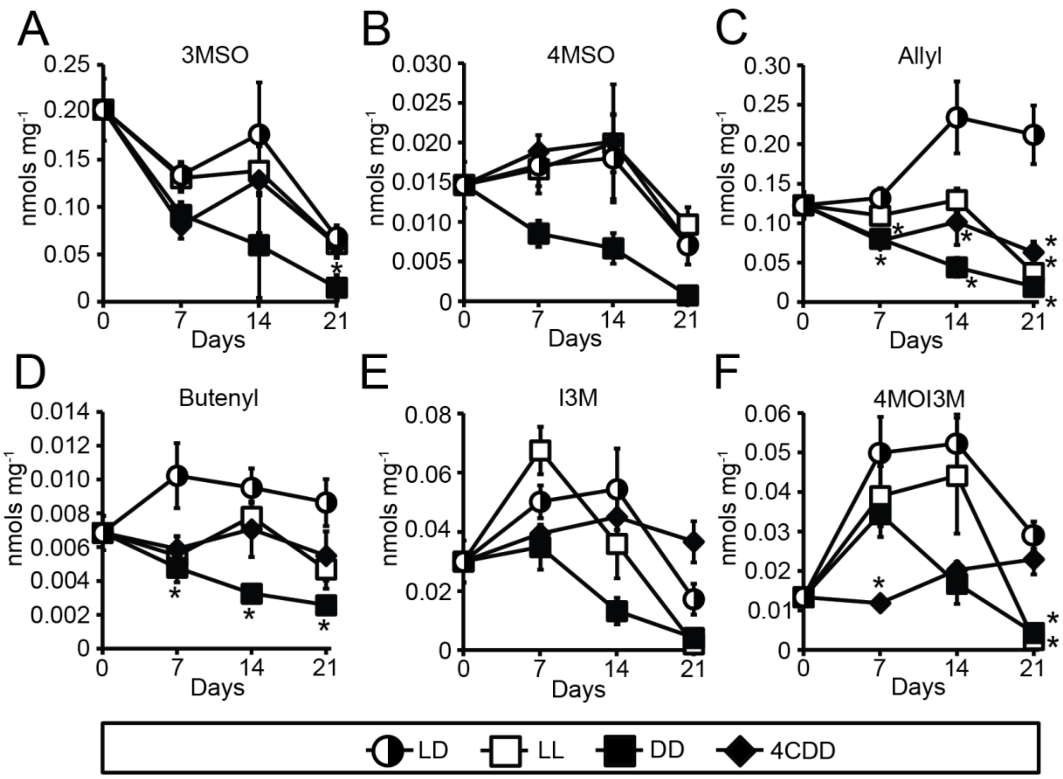

Figure S2. Accumulation of individual glucosinolate species in cabbage disks stored in 12-hour light/12-hour dark (22°C) (LD, half-filled circles), constant light (22°C) (LL, open squares); constant dark (22°C) (DD, filled squares), or refrigerated at 4°C in the dark (4CDD, filled diamonds). 3MSO, 3-methylsulfinylpropyl glucosinolate; 4MSO, 4-methylsulfinylbutyl glucosinolate; I3M, indoyl-3-methyl glucosinolate; 4MOI3M, 4-methoxy-indoyl-3-methyl glucosinolate. Mean ± SE; n = 4. Asterisks represent significant differences ($p<0.05$. ANOVA Test with Bonferroni Post Hoc analysis) between data derived from leaf disks stored under light/dark cycles (22°C) and that derived from leaf disks stored under other conditions at each time point.

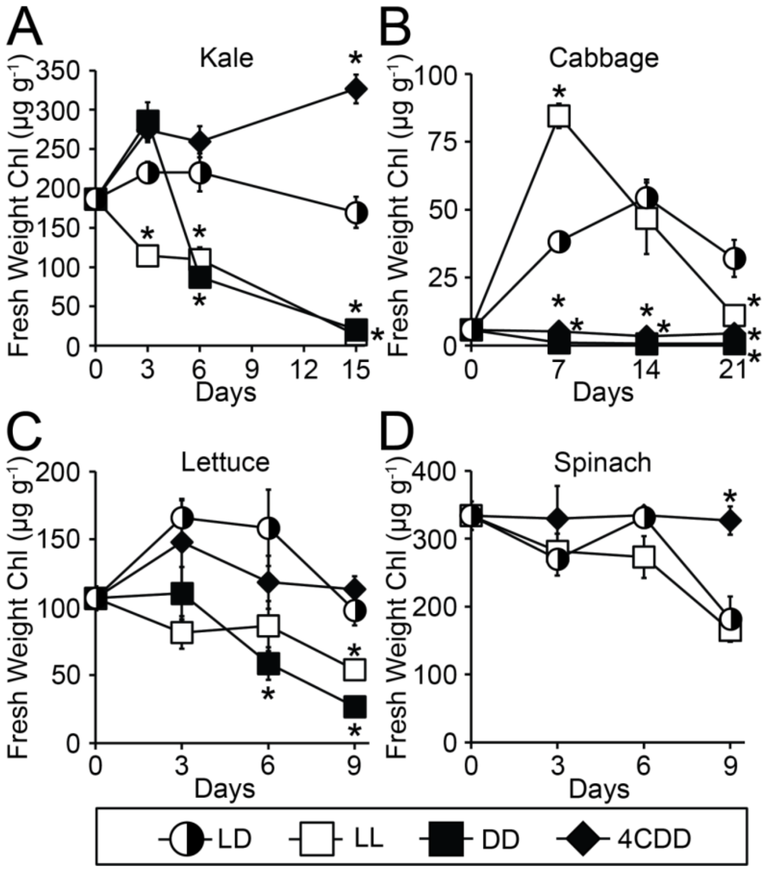

Figure S3. Chlorophyll content in fresh weight leaf tissue was maintained at higher levels in light/darkness stored vegetables. Chlorophyll content was quantified from leaf disk tissues of kale (A), cabbage (B), lettuce (C) or spinach (D) stored over time under cycles of 12-hours light/12-hours darkness (LD, half-filled circles), constant light (LL, open squares), or constant darkness (DD, filled squares) at 22°C, or under constant darkness at 4°C (4CDD, filled diamonds). Mean ± SE; n = 4. Asterisks represent significant differences ($p<0.05$. ANOVA Test with Bonferroni Post Hoc analysis) between data derived from leaf disks stored under light/dark cycles (22°C) and that derived from leaf disks stored under other conditions at each time point.

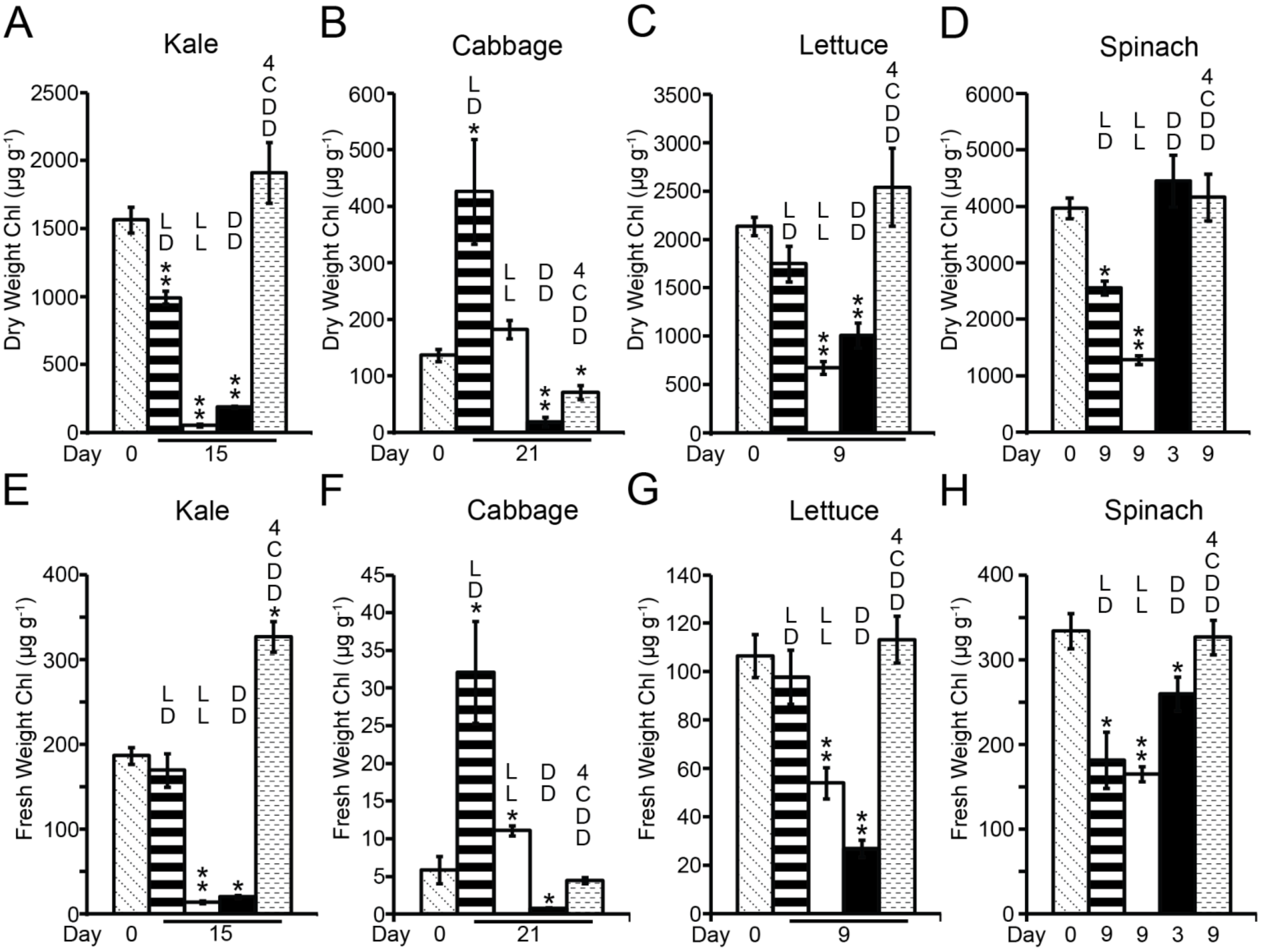

Figure S4. Chlorophyll content in fresh and dry weight leaf tissue was maintained at higher levels over time when stored in light/darkness cycles or under refrigeration. Chlorophyll content was quantified from dry weight leaf (A-D) and fresh weight (E-H) leaf disk tissues of kale (A, E), cabbage (B, F), lettuce (C, G) or spinach (D, H) stored over time under cycles of 12 hours light/12 hours darkness (LD), constant light (LL), or constant darkness (DD) at 22°C, or under constant darkness at 4°C (4CDD). Mean ± SE; n = 4. *p<0.05. **p<0.005. Unpaired student t-test analysis between day 0 concentrations and concentrations at the specified final time-point.

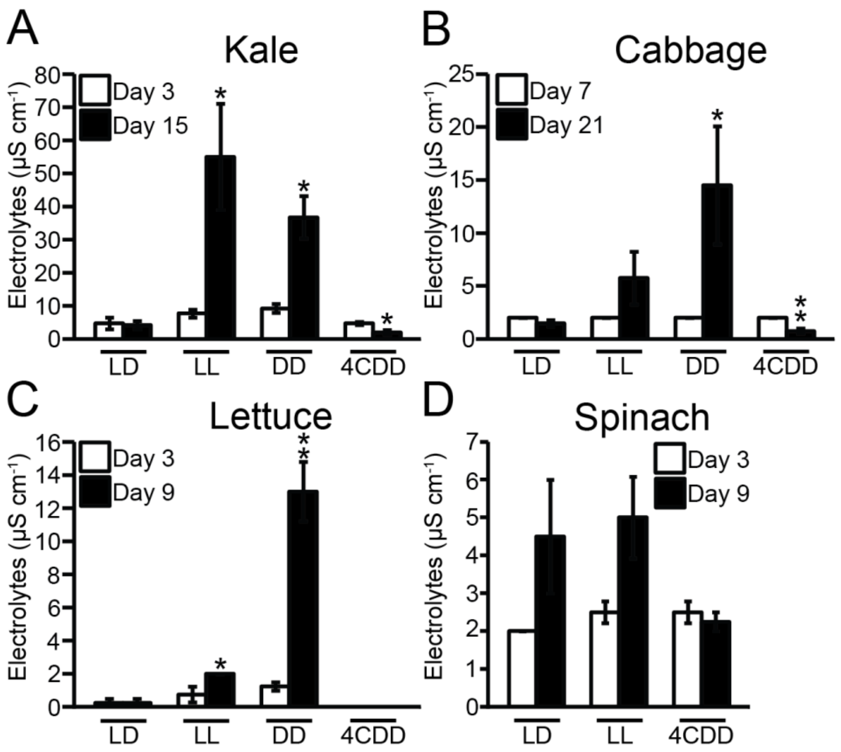

Figure S5. Electrolyte leakage from kale, cabbage, lettuce, and spinach leaf disks is increased when stored under constant light or constant dark in kale, cabbage and spinach. Electrolytes released from leaves of kale (A), cabbage (B), lettuce (C) or spinach (D) were measured after storage under cycles of 12-hour light/12-hour darkness (LD), constant light (LL), or constant darkness (DD) at 22°C or under constant darkness at 4°C (4CDD). Mean ± SE; n = 4. *p<0.05. **p<0.005. Unpaired student t-test analysis between measured initial levels and levels at the specified final time-point.

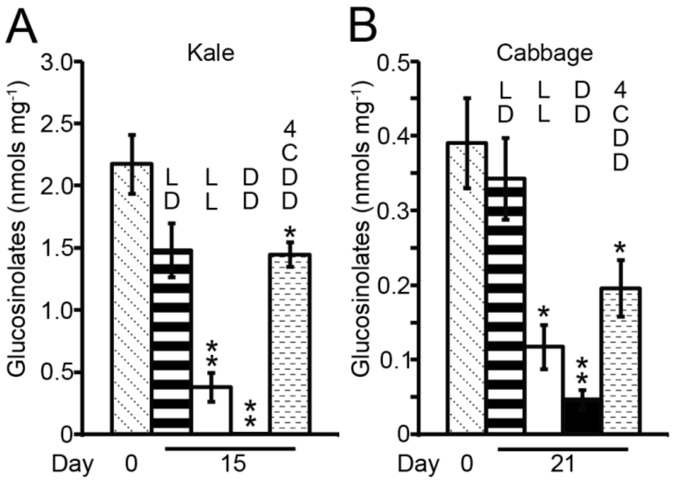

Figure S6. Maintenance of total glucosinolate levels in kale and cabbage leaves when stored under light/dark cycles. Glucosinolate species was quantified from leaf disks of kale (A) and cabbage (B) stored under cycles of 12-hour light/12-hour darkness (LD), constant light (LL), or constant darkness (DD) at 22°C or under constant darkness at 4°C (4CDD). Mean ± SE; n = 4. *p<0.05. **p<0.005. Unpaired student t-test analysis between day 0 concentrations and concentrations at the specified final time-point.
